# The Influence of Piezoelectric Transducer Stimulating Sites on the Performance of Implantable Middle Ear Hearing Devices: A Numerical Analysis

**DOI:** 10.3390/mi10110782

**Published:** 2019-11-14

**Authors:** Houguang Liu, Yu Zhao, Jianhua Yang, Zhushi Rao

**Affiliations:** 1 School of Mechatronic Engineering, China University of Mining and Technology, Xuzhou 221116, China; liuhg@cumt.edu.cn (H.L.); jianhuayang@cumt.edu.cn (J.Y.); 2 State Key Laboratory of Mechanical System and Vibrations, Shanghai Jiao Tong University, Shanghai 200240, China; zsrao@sjtu.edu.cn

**Keywords:** implantable middle ear hearing device, piezoelectric transducer, stimulating site, finite element analysis, hearing compensation

## Abstract

To overcome the inherent deficiencies of hearing aids, implantable middle ear hearing devices (IMEHDs) have emerged as a new treatment for hearing loss. However, clinical results show that the IMEHD performance varies with its transducer’s stimulating site. To numerically analyze the influence of the piezoelectric transducer’s stimulating sites on its hearing compensation performance, we constructed a human ear finite element model and confirmed its validity. Based on this finite element model, the displacement stimulation, which simulates the piezoelectric transducer’s stimulation, was applied to the umbo, the incus long process, the incus body, the stapes, and the round window membrane, respectively. Then, the stimulating site’s effect of the piezoelectric transducer was analyzed by comparing the corresponding displacements of the basilar membrane. Besides, the stimulating site’s sensitivity to the direction of excitation was also studied. The result of the finite element analysis shows that stimulating the incus body is least efficient for the piezoelectric transducer. Meanwhile, stimulating the round window membrane or the stapes generates a higher basilar membrane displacement than stimulating the eardrum or the incus long process. However, the performance of these two ideal sites’ stimulation is sensitive to the changes in the excitation’s direction. Thus, the round window membrane and the stapes is the ideal stimulating sites for the piezoelectric transducer regarding the driving efficiency. The direction of the excitation should be guaranteed for these ideal sites.

## 1. Introduction

Hearing loss, affecting around 466 million people worldwide, is one of the six leading causes of disease burden in our society [1]. Up to now, there is still no effective medical treatment to sensorineural hearing loss (SNHL), which is the main type of hearing loss taking up approximately 90% of reported hearing loss [2]. The patients with SNHL mainly turn to hearing aids for restoring audibility [3]. Although sophisticated hearing aids have been developed, hearing aids still have a number of inherent shortcomings, such as a limited high-frequency amplification gain, ear canal occlusion, and feedback annoyance [4]. To overcome these problems, many researchers proposed and designed the implantable middle ear hearing devices (IMEHDs), which restores audibility by the mechanical vibration of their implanted transducers [5].

**Figure 1:**
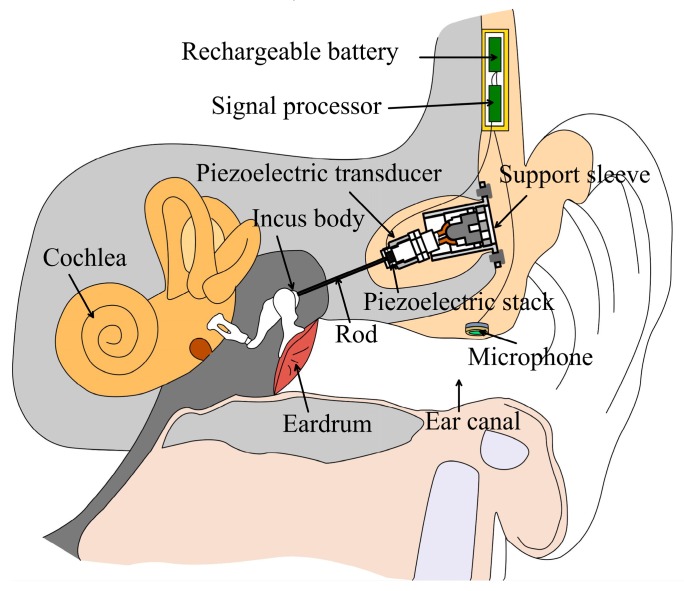
Schematic view of an implantable middle ear hearing device with a piezoelectric transducer attached on the incus body.

IMEHD primarily comprises four components: the microphone, the sound processor, the transducer, and the battery. A typical schematic illustration of the IMEHD is shown in Figure 1 [6]. Briefly, the microphone, which is implanted closer to the ear canal, receives the outside sound and transmits to the sound processor. Then, the sound processor processes the input signal according to patients’ hearing loss and outputs a driving signal to the piezoelectric transducer. The piezoelectric transducer mainly consists of three parts: the piezoelectric stack, the rod, and the support sleeve. One side of the piezoelectric stack is stuck to the rod, which is attached to the incus body. While the other side of the piezoelectric stack is held to the support sleeve, which is fixed to the skull. Under the electrical driving signal’s stimulation, the piezoelectric stack, which is a monolithic ceramic construction of many thin piezoelectric ceramic layers, expands and contracts. Finally, the vibration of the piezoelectric stack is transmitted to the incus body by the rod and compensates for hearing loss. All these parts are powered by the battery. Among these IMEHD parts, the transducer is a key component as it is responsible for stimulating the human ear. Based on actuation mechanisms, the IMEHDs’ transducers are divided into two types: the electromagnetic transducer and the piezoelectric transducer [5]. Compared with the electromagnetic transducer, the piezoelectric transducer has the advantages of a lower power consumption, compatibility with external magnetic field, and ease of fabrication [4]. Owing to these advantages, piezoelectric transducer have been widely used in IMEHDs, especially the totally implanted type IMEHDs [7]. In terms of the stimulating sites, the transducer can be further classified into five categories: the eardrum driving [8,9], the incus body driving [6], the incus long process driving [10], the stapes driving [11,12], and the round window (RW) membrane driving [13,14].

Clinical study shows that the stimulating site influence the transducer’s hearing compensation performance [15]. To uncover this influence and optimize the transducer’s design, some preliminary studies have been carried out. Based on numerical analysis, Zhang et al. found that stimulating the round window membrane is more efficient than stimulating the incus long process [16]. The human temporal bone experiment conducted by Deveze et al. demonstrates that stimulating the stapes is superior to stimulating the incus body and incus long process [17]. However, the above researches only focus on the electromagnetic transducer. A numerical study shows that the stimulating site’s influence on the electromagnetic transducer is different from that on the piezoelectric transducer [18]. To investigate the stimulating site’s effect on the piezoelectric transducer’s performance, Bornitz et al. constructed a human ear finite element (FE) model and compared the stapes displacements under different piezoelectric transducers’ stimulation [18]. Their result demonstrates that the incus body is the least effective stimulating site for the piezoelectric transducer. However, auditory response measurements show that the stapes response is unreliable for evaluating round window stimulation [19]. Besides this, stimulating the incus long process, which is widely utilized clinically, was not investigated.

Accordingly, in the present study, we carried out a systematic study on the influence of piezoelectric transducer’s stimulating sites. To facilitate this study, we built a human ear FE model and confirmed its validity. Then, the stimulating site’s effect was analyzed based on the basilar membrane’s displacement, which is reliable for evaluating IMEHD performance. The result could help the surgeon choose a piezoelectric transducer and aid the designer to optimize the piezoelectric transducer.

## 2. Materials and Methods 

### 2.1. Constructions of the Human Ear FE Model

**Figure 2:**
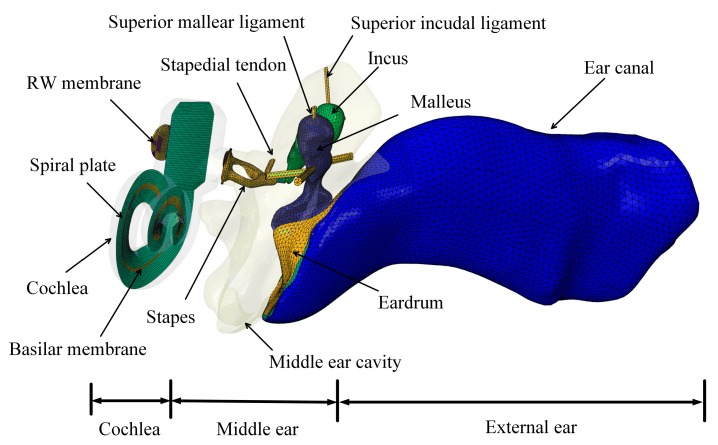
The constructed human ear finite element model.

A 3D FE model of the human ear was built using CT scanning and reverse modelling techniques based on a fresh human temporal bone specimen. Figure 2 shows the constructed model, consisting of the external ear canal, the middle ear (middle ear cavity, ossicular chain, and supporting ligaments and tendons), and the cochlea. The middle ear was separated from the external ear canal by the eardrum. The ossicular chain (malleus, incus, stapes and the joints) was connected to the wall of the middle ear cavity by the ligaments and tendons. The air in the middle ear cavity and the ear canal was meshed by acoustic tetrahedral elements, with a total of 277,863 elements. The eardrum was divided into the eardrum pars tensa and the eardrum pars flaccida. The eardrum pars tensa was established as a three-layer structure [20]. The inner layer and outer layer of the pars tensa was assumed to be isotropic, while the middle layer of the pars tensa was assumed to be orthotropic, with fibers in circumferential and radial directions. The eardrum pars tensa’s inner layer, middle layer, and the outer layer had a thickness of 0.017 mm, 0.016 mm, and 0.017 mm, respectively. The thickness of the eardrum annulus ligament and the eardrum pars flaccida were 0.2 mm and 0.1 mm, respectively. A total of 1939 three-noded shell elements were created to mesh the eardrum. The other middle ear structures were meshed by 45,609 four-noded tetrahedral elements.

The middle ear connects to the spiral cochlea with the stapes footplate attached to the oval window. The model’s cochlea consists of two fluid-filled chambers: the scala vestibuli (SV) and the scala tympani (ST). These chambers were separated by the basilar membrane (BM) and the bony spiral plate. A total of 361,589 four-noded acoustic tetrahedral elements were created to mesh the fluid in the cochlea. The BM and the bony spiral plate were meshed by 7666 shell elements. The BM thickness and width vary approximately linearly from the base of the cochlea to the apex of the cochlea. The BM length is 34 mm. The thickness of the BM varies from 5.2 μm to 0.6 μm, and the width varies from 0.1 mm to 0.5 mm. The round window membrane was meshed by 851 three-noded tetrahedral elements. The RW membrane has a thickness of 0.1 mm and an area of 2 mm^2^, which is close to the size of 2.08 mm^2^ reported by Atturo et al. [21].

Considering the ligaments and tendons connect to the bony wall of middle ear cavity, we fixed the end nodes of these components in our FE model. The surfaces of the acoustic elements, which attached to the bony wall in the ear canal, the middle ear cavity, and the cochlea, were defined as rigid walls. The outer edges of the round window membrane and the cochlear spiral plate were set as fixed constraints since they are anchored to the bony wall of the cochlea. Fluid structure interfaces were defined for the surfaces of the acoustic elements attached to the movable structures, i.e., the eardrum, the ossicles, the ligaments, the tendons, the oval window, the BM, and the round window membrane.

### 2.2. Material Properties

The middle layer of the eardrum pars tensa and the BM were assumed to be orthotropic. Other components of the FE model were assumed to be isotropic. Poisson’s ratios were assumed to be 0.3 for all components in the middle ear. The material properties of each component of the middle ear in the FE model were mainly referred to Gentil et al. et al. [20] and Zhang et al. [22], as listed in Table 1.

The components of the middle ear and the cochlea were modelled as elastic properties, except for the eardrum, eardrum annulus ligament, incudostapedial joint, incudomallear joint, stapedial annulus ligament, and RW membrane, which were modelled as linear viscoelastic materials. The Rayleigh damping was specified for the elastic components. The Rayleigh damping parameters were taken as *α* = 0 s^−1^, *β* = 0.0001 s [23]. The relaxation modulus of the linear viscoelastic materials was expressed as Equation (1):

(1) $$E(t)=E_{0}(1+e_{1}\exp(-\frac{t}{\tau_{1}}))$$

where *E*_0_, *e*_1_, and *τ*_1_ were viscoelastic parameters with constant values for each type of soft tissue, and *t* is the time. *E*_0_ is the elastic modulus listed in Table 1. *e*_1_, and *τ*_1_ are listed in Table 2. The viscoelastic parameters were referenced to Zhang et al.’s report [24]. These parameters were obtained by dynamic material tests on these components and the cross-calibration method.

The BM is assumed to be the anisotropic membrane with a density of 1200 kg/m^3^. The stiffness of BM was decreased from the base to the apex. The BM’s longitudinal modulus was assumed as 600 MPa at the base, linearly decreased to 10 MPa at the apex along the BM length. Similarly, the transverse modulus and the vertical modulus of the BM decrease linearly from 6 MPa, and 12 MPa at the base to 0.1 MPa, and 0.2 MPa at the apex, respectively. The density of the RW membrane was set to 1200 kg/m^3^ with an elastic modulus of 2.32 MPa [25]. The bulk modulus of the cochlear fluid and the air in the external ear canal and the middle ear cavity were set as 2250 MPa and 0.142 MPa, respectively. The viscosity of the cochlear fluid is 0.001 Ns/m^2^ [22].

### 2.3. Piezoelectric Transducer Simulation

**Figure 3:**
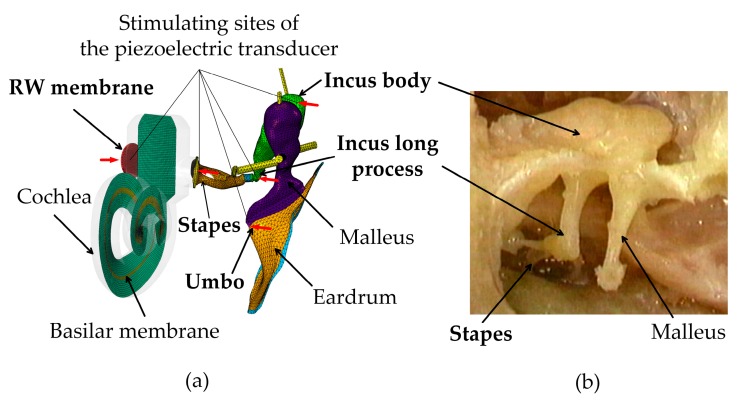
The simulation of the piezoelectric transducer’s simulation. (**a**) Stimulating sites on the finite element model; (**b**) the anatomy of the three ossicles (malleus, incus, and stapes).

Since the purpose of this paper is to study the stimulating site’s influence rather than the piezoelectric transducer’s structural design, we simplified the piezoelectric transducer as an ideal displacement-driven transducer. This idealized representation of the piezoelectric transducer is possible as small displacements and forces are required for hearing compensation in IMEHDs [18]. Based on this simplification, a displacement excitation with the magnitude of 0.1 μm was applied at the commonly used stimulating sites, i.e., the eardrum’s umbo, the incus long process, the incus body, the stapes, and the RW membrane, respectively. The magnitude of the applied displacement excitation was ascertained as it can produce a sound pressure level equivalent to 100 dB, which is a design criterion for an IMEHD transducer [4]. The stimulating sites were plotted in Figure 3. When stimulating the eardrum’s umbo, the incus long process, the incus body, the stapes, and the direction of the applied displacement excitation was along the longitudinal axis of the stapes, which is efficient for IMEHD stimulation [18]. For stimulating the round window membrane, the excitation’s direction was normal to the surface of the round window membrane. Under these forces’ stimulation, harmonic analysis was conducted over the frequency range of 0.25–6 kHz using the finite element software package ABAQUS (Dassault Systèmes, Johnston, RI, USA).

The surgical procedure, e.g., the transmastoidal approach for the piezoelectric transducer’s implantation, will possibly change the direction of the excitation. To study the stimulating site’s sensitivity to the direction changes of their excitations, the excitations were also applied in different directions at each stimulating site with the same magnitude of 0.1 μm. For stimulating the ossicular chain (umbo, incus long process, incus body, stapes), the reference direction was along the stapes’ longitudinal axis. The other directions are defined by rotating the direction relative to the reference direction in the plane based on the longitudinal axis and the long axis of the stapes’ footplate. The rotation is 20°, 45°, and 60° off the reference direction to crus posterior (20°, 45°, and 60° to CP). For stimulating the RW membrane, the reference direction is the normal direction of the RW membrane. The other directions are rotated 20°, 45°, and 60° off the reference direction.

### 2.4. Equivalent Sound Pressure Level

The sound transmission property via normal air conduction is different from that by a piezoelectric transducer’s stimulation. Considering the basilar membrane inside the cochlea is responsible for sensing the input vibration energy, we used its response to assess the transducer’s hearing compensation performance.

The vibration transmitted into the cochlea propagates in the form of a traveling wave from the base to the apex along the basilar membrane. For excitations of different frequencies, the maximum amplitude position of the traveling wave formed on the basilar membrane is different, with high frequencies maximally activating basal regions of the BM and low frequencies maximally activating apical areas of the BM. For a specific frequency excitation, the position of the basilar membrane that is most responsive in the longitudinal direction is referred to as the characteristic place of this frequency. The frequency is called the characteristic frequency of that position on the basilar membrane. The cochlea senses a pure tone sound of a specific frequency through its corresponding characteristic place along the basilar membrane. Therefore, in order to make the sound-perceived effect of the transducer’s stimulation of a specific frequency equivalent to that excited by normal sound stimulation (sound pressure applied at the eardrum), the displacements of the BM’s characteristic place of the frequency under the two excitations should be equal.

Based on above principle, in the normal sense of sound, when a sound with the frequency of *ω* and amplitude of *P*_E_ is applied at the eardrum, its stimulated BM displacement at the characteristic place $x_{\mathrm{CF}}$ is $d_{\mathrm{BM}}^{\mathrm{ac}}{(\omega ,x}_{\mathrm{CF}})$:

(2) $$d_{\mathrm{BM}}^{\mathrm{ac}}\left(\omega ,x_{\mathrm{CF}}\right)=TF_{d}^{\mathrm{ac}}\left(\omega \right)\cdot P_{E}$$

where $TF_{d}^{\mathrm{ac}}\left(\omega \right)$ is the transfer function of the normal human ear sensation from the pressure applied at the eardrum to the displacement of the basilar membrane. The human ear functions as a linear system under the normal acoustic sound pressure excitation [26]. Therefore, based on the model-calculated basilar membrane’s displacement under 100 dB SPL sound stimulation applied at the eardrum, we can obtain the transfer function:

(3) $$TF_{d}^{\mathrm{ac}}\left(\omega \right)=\frac{d_{\mathrm{BM}}^{{\mathrm{ac}}_{100}}\left(\omega ,x_{\mathrm{CF}}\right)}{2\times 10^{-5}\times 10^{\frac{100}{20}}}.$$

Under the excitation of the ideal piezoelectric transducer, its stimulated BM displacement at the characteristic place $d_{\mathrm{BM}}^{\mathrm{piezo}}\left(\omega ,x_{\mathrm{CF}}\right)$ can be calculated by the FE model. Since the basilar membrane vibration is responsible for transmitting the input energy to hair cells, the transducer-stimulated effect is equivalent to that excited by a normal acoustic stimulation ${\overset{⏜}{P}}_{E}$ applied at the eardrum, which produces the same displacement amplitude ${\overset{⏜}{d}}_{\mathrm{BM}}^{\mathrm{ac}}\left(\omega ,x_{\mathrm{CF}}\right)$ at the characteristic place of the basilar membrane:

(4) $$d_{\mathrm{BM}}^{\mathrm{piezo}}\left(\omega ,x_{CF}\right)={\overset{⏜}{d}}_{\mathrm{BM}}^{\mathrm{ac}}\left(\omega ,x_{\mathrm{CF}}\right)=TF_{d}^{\mathrm{ac}}\left(\omega \right)\cdot {\overset{⏜}{P}}_{E}=\frac{d_{\mathrm{BM}}^{{\mathrm{ac}}_{100}}\left(\omega ,x_{\mathrm{CF}}\right)}{2\times 10^{-5}\times 10^{\frac{100}{20}}}\cdot {\overset{⏜}{P}}_{E}.$$

Based on Equation (4), the transducer’s corresponding equivalent sound pressure ${\overset{⏜}{P}}_{E}$ applied at the eardrum can be derived as

(5) $${\overset{⏜}{P}}_{E}=\frac{d_{\mathrm{BM}}^{\mathrm{piezo}}\left(\omega ,x_{CF}\right)}{d_{\mathrm{BM}}^{\mathrm{ac}\_100}\left(\omega ,x_{\mathrm{CF}}\right)}\times 2\times 10^{-5}\times 10^{\frac{100}{20}}.$$

Thus, the performance of the transducer’s excitation can be evaluated by $L_{\mathrm{EQ}}$, which is the equivalent sound pressure level (ESPL) of the piezoelectric transducer:

(6) $$L_{\mathrm{EQ}}=20\log\frac{{\overset{⏜}{P}}_{E}}{2\times 10^{-5}}=100+20\log(\frac{d_{\mathrm{BM}}^{\mathrm{piezo}}\left(\omega ,x_{\mathrm{CF}}\right)}{d_{\mathrm{BM}}^{\mathrm{ac}\_100}\left(\omega ,x_{\mathrm{CF}}\right)}).$$

## 3. Results

### 3.1. Validation of the Human Ear Finite Element Model

**Figure 4:**
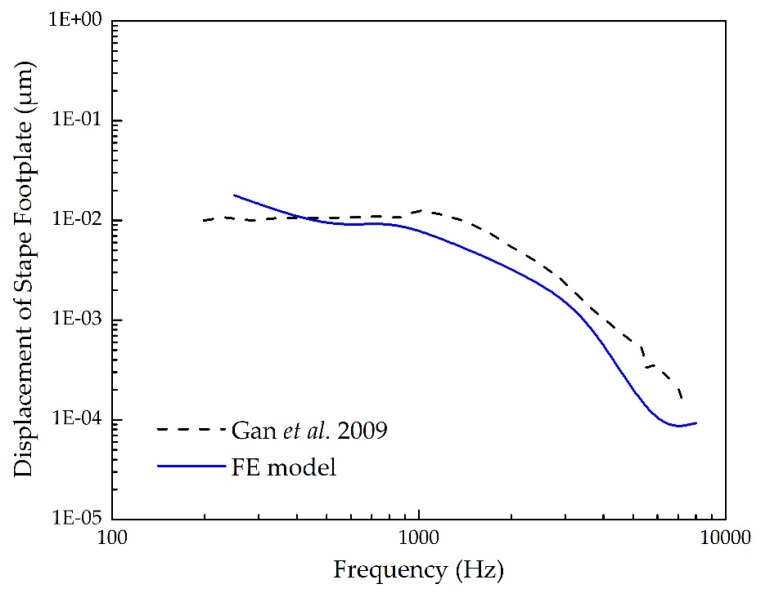
Comparison of the stapes’ footplate displacement under 90 dB SPL sound pressure applied at the eardrum.

To confirm the validity of the established human ear finite element model, three sets of comparisons with the published experimental data were conducted. Since the stapes response is the input of the cochlea, we firstly selected the stapes’ footplate displacement to verify our model. Figure 4 shows the mean value of experimental measurements on five temporal bones reported by Gan et al. [27]. In this experiment, a set of pure tone sounds of 90 dB SPL were applied to the eardrum, and the displacement of the stapes footplate was measured using a laser vibrometer. For comparison, we carried out a harmonic analysis across the frequency range of 250–8000 Hz under the same sound pressure applied to the lateral side of the eardrum of our FE model. The model-predicted result was also plotted in Figure 4. It demonstrates that our model-derived displacement of the stapes footplate agrees well with the experimental curve.

**Figure 5:**
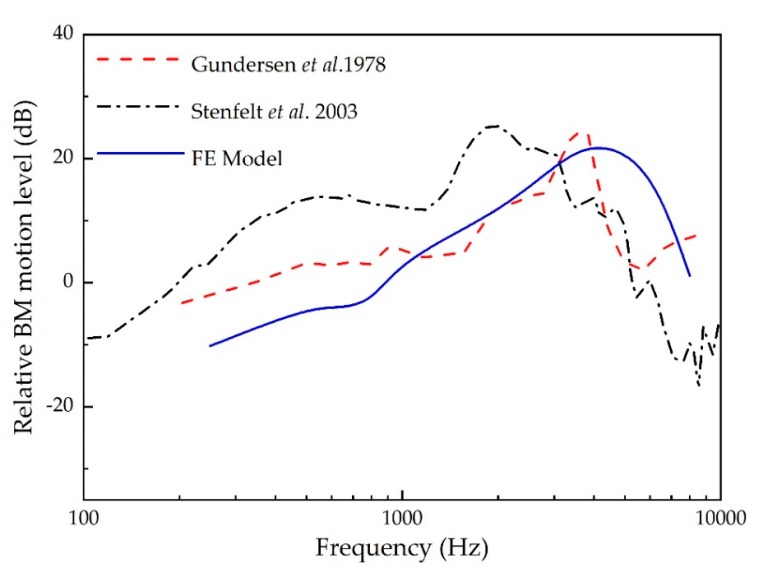
Comparison of the ratio of the basilar membrane’s (BM) velocity at 12 mm from the stapes to the stapes’ velocity.

The BM’s response was also selected for our model’s validation as it responsible for sensing the cochlear input vibration. Figure 5 displays the experimental curves of the ratio of the BM’s velocity at 12 mm from the stapes to the stapes’ velocity. The experimental tests were conducted by Gundersen et al. [26] and Stenfelt et al. [28] with a 90 dB SPL input sound pressure applied to the eardrum. Similarly, with a uniform sound pressure applied at the lateral side of the eardrum in our model, a harmonic analysis was conducted across the frequency range of 250–8000 Hz. The model-calculated result was plotted with the experimental curves in Figure 5. It shows that the maximum peak appears at 3500 Hz, which conforms to the experimental data of Gundersen et al. [26]. Besides, our model-predicted result has the same trend as Stenfelt et al.’s [28] data.

**Figure 6:**
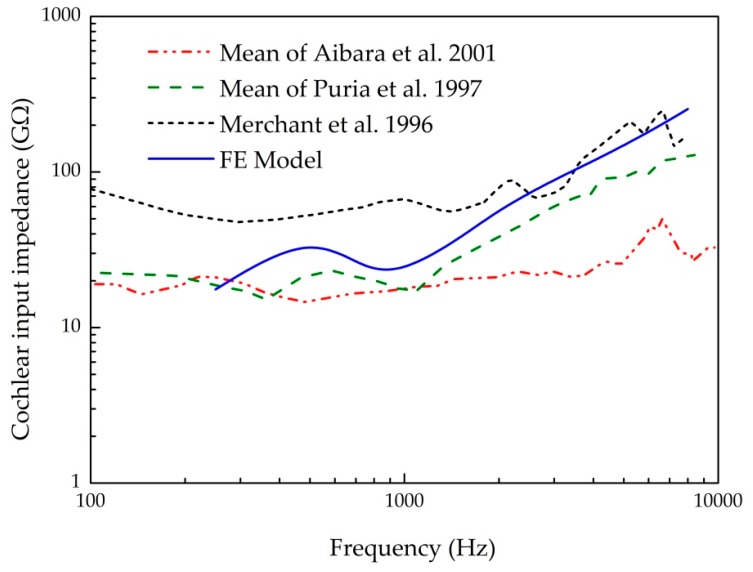
Comparison of the cochlear input impedance.

Finally, we compared the model-derived cochlear input impedance, which is a measure to represent the cochlear resistance of transmitted vibration from the middle ear, with the experimental data measured by Aibara et al. [29], Puria et al. [30], and Merchant et al. [31], as shown in Figure 6. The cochlear input impedance was calculated from the ratio of the pressure in the SV to the stapes volume velocity (product of the stapes’ footplate velocity and the stapes’ footplate area). It shows that our predicted result is in the range of these experimental data, and has the same trend with these experimental data, especially the data of Puria et al. [30]. These above comparisons prove that our model can be utilized to predict the sound transmission properties of the human ear.

### 3.2. The Stimulating Site’s Influence on the Piezoelectric Transducer’s Performance

**Figure 7:**
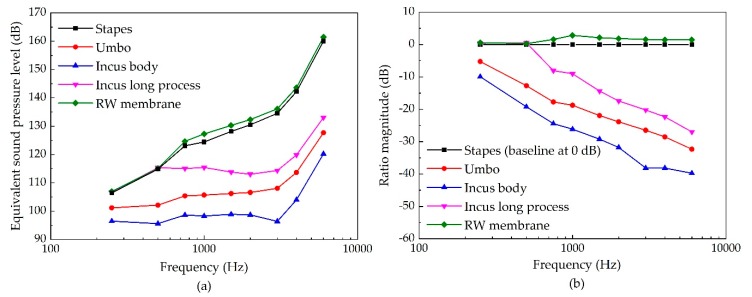
The influence of a piezoelectric transducer’s stimulating sites on its hearing compensation performance. (**a**) Equivalent sound pressure level of the piezoelectric transducers stimulating at different sites; (**b**) ratio of equivalent sound pressure of the piezoelectric transducer stimulating at different sites (the reference is the stimulation applied at the stapes along the stapes longitudinal axis).

Figure 7 shows the influence of the piezoelectric transducer’s stimulating sites on its hearing compensation performance. It demonstrates that the piezoelectric transducer can produce high ESPL at high frequency no matter which sites is stimulated. Stimulating the RW membrane as well as stimulating the stapes can produce a more equivalent sound pressure level than stimulating the other sites, especially at a high frequency. Besides, the ESPL under the stimulation applied at the incus-long-process is superior to that generated by the umbo stimulation. The incus body is the worst stimulating site for the piezoelectric transducer.

### 3.3. The Sensitivities of Each Stimulating Site to the Changes of Excitation’s Direction

**Figure 8:**
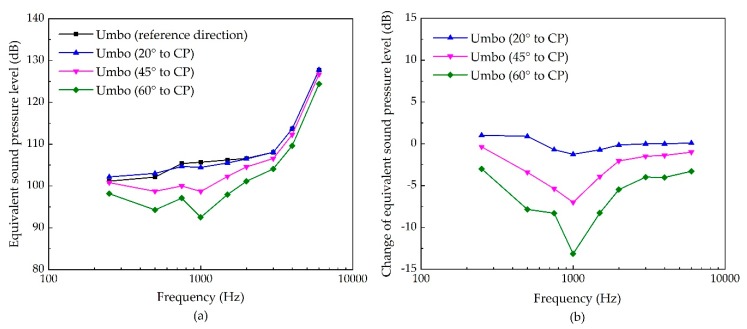
The sensitivity of umbo stimulation due to the change of its excitation’s direction. (**a**) Equivalent sound pressure level of the piezoelectric transducer’s stimulation; (**b**) change of equivalent sound pressure level.

For stimulating the eardrum’s umbo, the influence of a piezoelectric transducer’s excitation direction on its hearing compensation performance is shown in Figure 8. It indicates that the transducer’s stimulated ESPL decreases with the increase of the angle of the excitation’s inclination, especially at the middle frequency. The maximum decrease is found at 1 kHz for 60° to CP, with a reduction of 13 dB.

**Figure 9:**
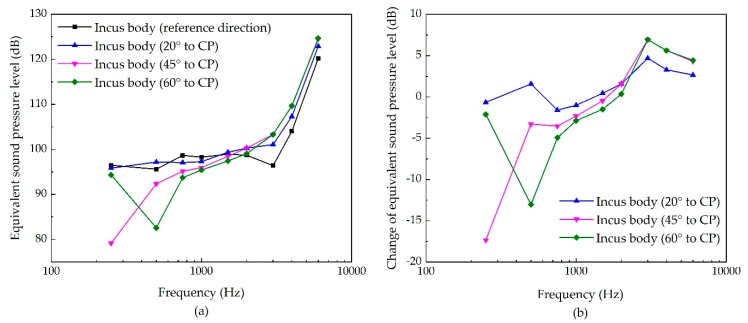
The sensitivity of incus-body stimulation due to the change of its excitation’s direction. (**a**) Equivalent sound pressure level of the piezoelectric transducer’s stimulation; (**b**) change of equivalent sound pressure level.

While the stimulating site is the incus body, the stimulation direction’s influence is shown in Figure 9. It demonstrates that the change of the stimulation direction’s influence on the transducer’s stimulated ESPL is complex in this case. Increasing the angle of the stimulation’s inclination decreases the transducer-stimulated ESPL at a lower frequency, but increases the ESPL slightly at a higher frequency. The maximum decrease is at 250 Hz for 45° to CP, with a reduction of 17 dB.

**Figure 10:**
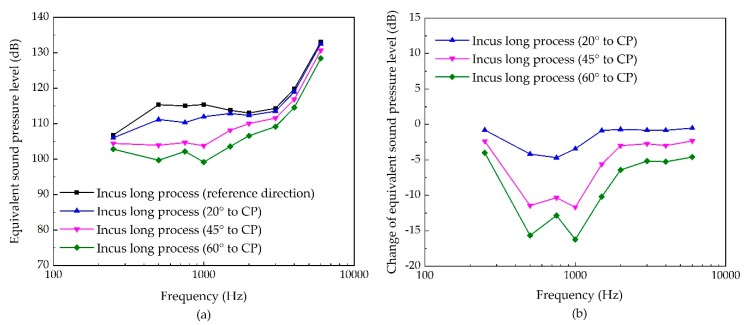
The sensitivity of incus-long-process stimulation due to the change of its excitation’s direction. (**a**) Equivalent sound pressure level of the piezoelectric transducer’s stimulation; (**b**) change of equivalent sound pressure level.

As for stimulating the incus long process, the excitation direction’s influence is shown in Figure 10. Similar to the influence in stimulating the eardrum’s umbo, the boost of the angle of the excitation’s inclination reduces the transducer-stimulated ESPL, especially at the middle frequency. The maximum decrease is also at 1 kHz for 60° to CP, with a reduction of 16 dB.

**Figure 11:**
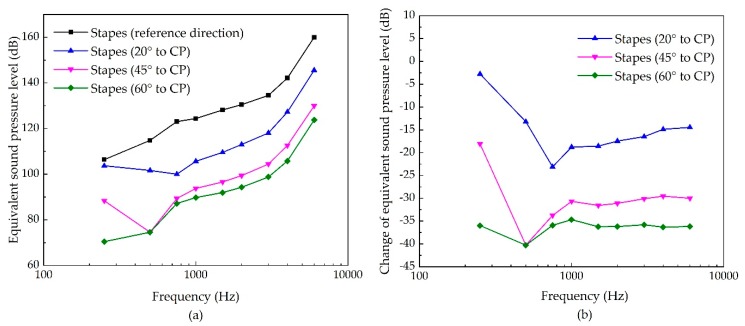
The sensitivity of stapes stimulation due to the change of its excitation’s direction. (**a**) Equivalent sound pressure level of the piezoelectric transducer’s stimulation; (**b**) change of equivalent sound pressure level.

In terms of stimulating the stapes, the effect of the transducer’s stimulation direction is shown in Figure 11. It indicates that the transducer-stimulated ESPL also decreases with the increase of inclination angle. Unlike previous stimulating sites, the ESPL at a high frequency decreases significantly in this case. The maximum decrease is at 400 Hz for 60° to CP, with a reduction of 40 dB. For a high frequency region, the maximum reduction is 36 dB at 4 kHz for 60° off the reference direction.

**Figure 12:**
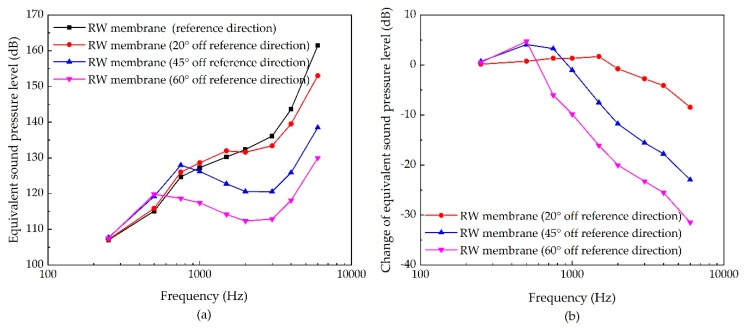
The sensitivity of RW membrane stimulation due to the change of its excitation’s direction. (**a**) Equivalent sound pressure level of the piezoelectric transducer’s stimulation; (**b**) change of equivalent sound pressure level.

For stimulating the RW membrane, the influence of the transducer’s stimulation direction is shown in Figure 12. It demonstrates that the increase of the inclination angle mainly reduces the transducer’s high frequency ESPL. The maximum decrease is at 6 kHz for 60° off the reference direction, with a reduction of 31 dB.

## 4. Discussion

Since the human ear is a complicated biological system with tiny structures and complex geometry, systematic experimental investigation on it is tough to conduct. Considering the finite element method has the advantage of simulating this complicated biological system, many researchers built a human ear FE model, and used it to study the sound transmission properties of the ear [32,33,34,35] and facilitate the design of IMEHDs [14,36,37]. In our human ear FE model, the cochlea was constructed as two fluid-filled channels. This modelling method of the cochlea is widely used in the field of cochlear mechanics [16]. Different individual human ears have similar vibration properties, and to confirm our model’s validity, we compared our model-predicted results with the mean value of the experimental results, which were measured on many human ears, to prove that our model can predict the general response of the human ear. This kind of validation has been widely used by scholars in this fields [20,23].

**Figure 13:**
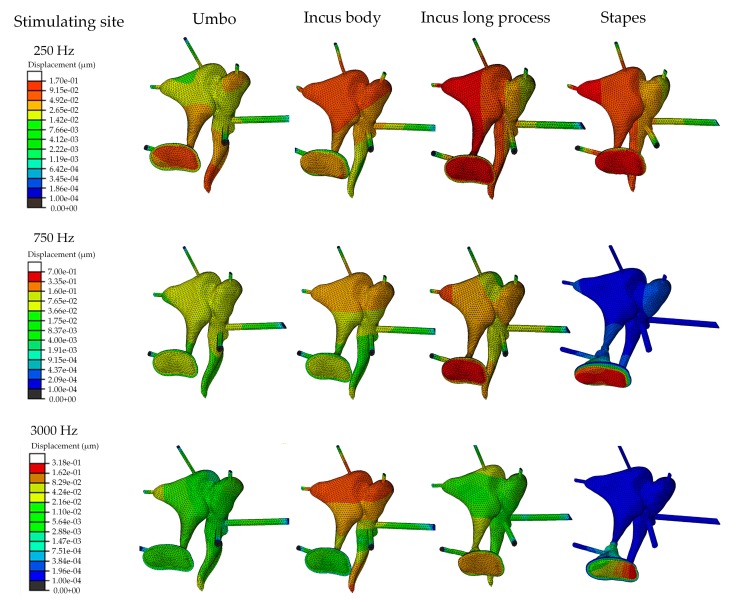
The z-direction (along the longitudinal axis of the stapes) displacement contour plot of the ossicular chain.

According to the vibrational energy transmission pathway, the implantable middle ear hearing device can be classified as forward stimulation and reverse stimulation [38]. Stimulating the eardrum, the incus body, the incus long process, and the stapes belong to forward stimulation, since their vibration energy are transmitted to the cochlea through the cochlear oval window, which is the same as the normal hearing process. Whereas, stimulating the round window membrane is called reverse stimulation as its vibration energy is transmitted to the cochlea though the cochlear round window, the other opening window of the cochlea. For the forward driving, our results demonstrate that the piezoelectric transducer provides better performance when stimulating the stapes than stimulating the eardrum’s umbo or the incus long process. The performance of stimulating the incus long process is superior to that of stimulating the eardrum’s umbo. This result can be easily predicted for forward stimulation since the stapes is close to the cochlea and therefore more efficient to transmit vibrational energy into the cochlea. Besides, we found that the superiority of the stapes stimulation is significant at high frequencies. To further analyze this phenomenon, we plot the z direction’s (along the longitudinal axis of the stapes) displacement contour plot (Figure 13) of the ossicular chain since the stapes transmits its vibration mainly through its piston motion [39]. Figure 13 shows that the stapes can be efficiently stimulated at a low frequency for all these three stimulating sites, especially for stimulating the stapes and stimulating the incus long process. With an increase in the stimulation’s frequency, the vibration cannot be effectively transmitted to the stapes for stimulating the incus long process and the umbo, especially for stimulating the umbo. This may attribute to the incudomallear joint and the incudostapedial joint, whose viscous behavior become significant at a higher frequency and weaken the vibrational energy transmission from the stimulating point to the stapes. 

For the forward stimulation, the incus body is the worst stimulating site for the transducer. This result is consistent with Bornitz et al.’s report [18] based on stapes displacement. This owing to the fact that the rotation nod of the ossicular chain is close to the incus body [40]; therefore, the incus body is the least efficient point for stimulating the ossicular chain. Figure 13 also shows that most of the stimulated response are restrained around the incus body; the vibration cannot be efficiently transmitted to the stapes footplate under the incus-body stimulation, especially at a high frequency. Thus, compared with other forward stimulation, the high-frequency output should be enhanced for the incus-body simulating-type piezoelectric transducer.

Compared with forward stimulation, it is difficult to estimate the performance of the round window’s stimulation, i.e., the reverse stimulation, since its vibration energy transmission pathway is different from that of forward driving. Although comparison of the forward stimulation with the round window’s stimulation were reported [16,22], these studies only focus on the electromagnetic transducer, which is a force-driven transducer. Bornitz et al.’s study [18] demonstrates that the stimulating site’s influence for the electromagnetic transducer is different from that for the piezoelectric transducer. For the piezoelectric transducer, our study finds that stimulating the round window membrane can produce a similar ESPL as when stimulating the stapes. Regardless of which site is stimulated, the piezoelectric transducer can generate high ESPL at a high frequency. Since most sensorineural hearing loss is severe at a high frequency [41], this characteristic is a crucial advantage for the piezoelectric transducer to compensate for the hearing loss. This better high-frequency performance of the piezoelectric transducer was reported in many experimental studies [6,10,42]. 

The performance of the RW membrane stimulation, as well as that of the stapes stimulation are susceptible to the change of the excitation’s direction. This result for the RW membrane stimulation conforms to Arnold et al.’s temporal bone’s study [43], which found that the transducer’s direction significantly affects the energy transferred to the cochlea of up to 35 dB. The clinical result also shows that the postoperative performance of the RW stimulation has a high variability [44], which may attribute to the change of the transducer’s direction. Meanwhile, this sensitivity for RW membrane stimulation and stapes stimulation to the excitation direction is prominent at a high frequency. Considering the main type of sensorineural hearing loss is the “high-frequency” hearing loss [41], the piezoelectric transducer’s orientation for RW stimulation or stapes stimulation should be guaranteed during the surgery. For the design of these two types of transducers, it is recommended to design a fixing part to ensure its orientation after implantation.

The main purpose of this paper is to investigate the stimulating site’s influence on the piezoelectric transducer. To facilitate this study, the real structure of the piezoelectric transducer was not considered in this paper; instead, we simplified it as an ideal displacement driven transducer, i.e., a transducer generates a certain displacement without limitations in force. Under this simplification, the retroaction of the human ear system onto the piezoelectric transducer was neglected. This simplification for the IMEHD’s piezoelectric transducer is based on the fact that the blocking force of the transducer is much larger than its working force. For instance, the piezoelectric transducer (Model PL-033, Physik Instrumente, Waldbronne, Germany) used in Wang et al.’s study [41] for IMEHD has a blocking force of 300 N, which is much larger than the force (89 µN [45]) required to drive the vibration of ossicles to the equivalent of 100 dB SPL. Thus, the resistant force of the human ear cannot change the piezoelectric displacement output significantly. Based on a coupled FE model of the middle ear and a piezoelectric transducer, which is a 20-layer stack of 3 mm diameter and 4 mm thickness made of PZN-4.5PT, Bornitz et al. [18] also found that there is no retroaction of the human ear onto the piezoelectric transducer. Thus, simplifying the piezoelectric transducer in IMEHD as a displacement-driven transducer is acceptable.

It should be noted that our FE model is constructed only based on one human ear specimen. Based on a numerical study, Daniel et al. [46] found that the human ear’s geometrical variation can lead to differences of 4 dB in the lower frequencies and up to 6 dB around 2 kHz, but similar shapes in the calculated response curves. Thus, the patients’ individual geometrical differences may alter our results quantitatively at lower frequencies and frequencies around 2 kHz. Nevertheless, the overall trend of our results still holds under different individual human ear geometries.

## 5. Conclusions

To study the influence of piezoelectric transducer’s stimulating sites on its hearing compensation performance, a human ear FE model, including the ear canal, the middle ear, and the cochlea, was constructed. The validity of this model was verified by three sets of comparisons. The results show the piezoelectric transducer provides better performance when simulating the stapes or RW membrane than stimulating other studied sites, especially at a high frequency. The incus body is the worst stimulating site for the piezoelectric transducer. Meanwhile, the performance of the RW membrane stimulation, as well as that of the stapes stimulation, are susceptible to the change of the excitation’s direction. Considering most sensorineural hearing loss is severe at high-frequency, the piezoelectric transducer’s orientation for RW stimulation or stapes stimulation should be guaranteed during the surgery.

## Figures and Tables

**Table 1:** Material properties of the middle ear components.

Components	Layer	Young’s Modulus (N/m^2^)	Density (kg/m^3^)
Eardrum annulus ligament		2.00 × 10^5^	1.20 × 10^3^
Eardrum pars tensa	Outer layer	1.00 × 10^7^	1.20 × 10^3^
	Middle layer	*E_θ_* = 2.00 × 10^7^, *E_r_* = 3.20 × 10^7^	
	Inner layer	1.00 × 10^7^	
Eardrum pars flaccida		1.00 × 10^7^	1.20 × 10^3^
Malleus handle		1.41 × 10^10^	3.70 × 10^3^
Malleus neck		1.41 × 10^10^	4.53 × 10^3^
Malleus head		1.41 × 10^10^	2.55 × 10^3^
Incus body		1.41 × 10^10^	2.36 × 10^3^
Incus short process		1.41 × 10^10^	2.26 × 10^3^
Incus long process		1.41 × 10^10^	5.08 × 10^3^
Stapes		1.41 × 10^10^	2.20 × 10^3^
Incudomallear joint		6.00 × 10^7^	3.20 × 10^3^
Incudostapedial joint		2.00 × 10^6^	1.20 × 10^3^
Lateral malleolar ligament		6.70 × 10^6^	2.50 × 10^3^
Superior malleolar ligament		4.90 × 10^6^	2.50 × 10^3^
Anterior malleolar ligament		8.00 × 10^6^	2.50 × 10^3^
Posterior incudal ligament		6.50 × 10^6^	2.50 × 10^3^
Superior incudal ligament		4.90 × 10^6^	1.00 × 10^3^
Tensor tympani tendon		8.00 × 10^6^	2.50 × 10^3^
Stapedial tendon		5.20 × 10^7^	1.00 × 10^3^
Stapedial annulus ligament		1.00 × 10^4^	1.20 × 10^3^

**Table 2:** Parameters of linear viscoelastic materials.

Components	*e*_1_	*τ*_1_ (μs)
Eardrum annulus ligament	3.2	28
Eardrum pars flaccida	2.29	25
Eardrum pars tensa	2.8	25
Incudomallear joint	3.0	20
Incudostapedial joint	50	20
Stapedial annulus ligament	2.4	25
RW membrane	3.0	30
